# 

**Published:** 1841-09

**Authors:** 


### CONTENTS

OF NO. III.

### ORIGINAL COMMUNICATIONS.

### ESSAYS AND CASES.

Art. I.—An Essay on Bilious Fever. By Lunsford P. Yandell, M. D. - - - - - - 161

### SELECTIONS FROM AMERICAN AND FOREIGN JOURNALS.

Report of a Case of Axillary Aneurism, in the General Hospital, U. S. A., at Picolata, E. F. - - - - 205
Compression of the Aorta as a means of arresting Uterine Hemorrhage. - - - - - - - 209
Clinical Lecture on Fissure of the Anus. - - - 216
Treatment of Rheumatism by Inoculation with Morphia. 224
Extraction of a Foreign Body implanted in the Uterus. 225

### ORIGINAL INTELLIGENCE.

Prize Essay. - - - - - - - 227
Medical Schools. - - - - - - 228
Medical Institute of Louisville. . - - - - 228

Stramonium in Neuralgic Affections. - - - 229
Treatment of Milk-Sickness. - - - - - 229
Symptoms of Poisoning with Whiskey. - - 230
Unpublished Contributions. - - - - 230
Resistance of the Liver to Putrefaction. - - 232
Worthy of Imitation. - - - - - 232
Orthopœdic Institution—Prof. Mott. - - - 233
Morbid Anatomy of Milksickness. - - - 234
Clerical Encouragement of Quackery. - - - 235
Stammering. - - - - - - 236
Medicine in Paris. - - - - - 237

